# A Professor on Tour

**Published:** 1898-12-10

**Authors:** 


### A PROFESSOR ON TOUR.

When a great physician takes a tramp abroad he has to beware of many things, and among them has to look out for the medical reporter; not the faithful and laborious stenographer, but the sketchy gentleman who gives a lecture in half a dozen paragraphs, and condenses a life's work into a few jerky sentences. Professor Clifford Allbutt seems to have been relieving the tedium of travel by speechification, and among other discourses he has given a lecture at Jefferson Medical College on the "Therapeutic Aspect of Circulatory Disturbances," out of which the reporter for the *International Medical Magazine* has picked some gems which he seems to have reported in his own peculiar manner. "Really, therapeutics is a small matter, and easily disposed of," Professor Allbut is made to say, "if you once make the diagnosis of the disease. Diagnosis is the great thing. In fact, therapeutics is only diagnosis. After this is made the rest is easy. In fact, you may not use drugs at all when once you know what ails your man. You may prescribe gymnastics, massage, air, water, electricity. You set the forces of nature at work repairing another part." . . . . "Study processes and combat them. Do not wait to make the diagnosis until the final stage begins which will lead to an incurable disease. You should know every detail as to how organs are doing their work. See just how the liver, the kidneys, the lungs are doing. Modern therapeutics simply means prevention. There are no specifics for diseases any more—the disease cures itself." . . . And so on for a couple of pages, ending with the following exordium: "So we see that therapeutics is physiology. It is pathology. It is not writing a prescription for a certain disease. First-class therapeutics is studying and combating diseased processes. It is preventive. But if you cannot take a first-class course in dealing with disease, do the next best you can. Do something. If you cannot locate processes or diagnose causes and cure them, take a second-class course and give what has been customary. It may do good. You must do something for your patient, and the mere taking of medicine has in it a great element of suggestion for most people." And so the Professor wanders round the world, pouring out his wisdom in short aphorisms, as if out of his spacious pockets he were throwing small coin to the multitude.

